# The characteristics and comparison between young and old patients with spontaneous isolated celiac artery dissection: Analysis based on 60 reports

**DOI:** 10.1097/MD.0000000000036255

**Published:** 2023-12-08

**Authors:** Xiaoliang Chen, Hongtao Wu, Shenghuan Wang, Tianbo Feng

**Affiliations:** a Department of Traumatology Surgery, Gansu Provincial Qingyang People’s Hospital, Qingyang, China.

**Keywords:** characteristic, isolated celiac artery dissection, management

## Abstract

**Background and aims:**

Though increasing studies reported the management of spontaneous isolated celiac artery dissection (ICAD), the characteristics and etiology of ICAD in different age-stage patients have not been well-studied. Our study was designed to describe and further to compare the clinical features of spontaneous ICAD between young and old patients.

**Methods:**

We searched PubMed, Embase, and Web of Science up to March 1, 2023 for spontaneous ICAD case reports. Two reviewers screened the titles and abstracts of searched records for qualified reports according to the including and excluding criteria and extracted the data independently. Statistical analysis was performed using SPSS software (version 19.0; IBM Corp, Armonk, NY) and Stata 12.0 (Stata Corp., College Station, TX). Descriptive results were presented as the mean ± standard deviation or percent. The comparison results between young and old patients were displayed as risk ratio (RR) or standardized mean difference (SMD) with its 95% confidence intervals (CI).

**Results:**

We totally identified 60 reports in the present analysis. The mean age of patients was 52.4 years, with the majority of patients being male (84.4%). The majority of patients were symptomatic and commonest presentation was abdominal pain (76.7%). Most patients (63.2%) had comorbidities or history and hypertension and smoking were the top 2 conditions with proportion of 63.3% and 40.5% respectively. When comparing young to old patients with ICAD, no significant difference was found in demographic and clinical features including sex, comorbidities/history, and symptoms. However, we found that young patients with ICAD experienced significantly longer dissection length (SMD 1.01, 95% CI 0.16–1.86; *P* = .015) and distance from ostium (SMD 0.96, 95% CI 0.07–1.85; *P* = .013), but no significant difference was observed in true lumen compression (SMD −0.39, 95% CI −1.22–0.44; *P* = .364). In addition, our results showed that ICAD in young patients extending more to distal arteries, including common hepatic artery/hepatic artery (RR 2.04, 95% CI 1.13–3.68; *P* = .01), splenic artery (RR 2.36, 95% CI 1.24–4.49; *P* = .017) and left gastric artery (RR 25.42; 95% CI 1.55–417.74; *P* = .04).

**Conclusions:**

Though spontaneous ICAD had multitudinous clinic-pathologic features, it was apt to middle-aged males and symptomatic and abdominal pain was always the commonest presentation. Hypertension and smoking were the top 2 conditions of ICAD patients. There was significant difference between young and old patients in radiographic characteristics of ICAD which may lead to different treatment and outcomes.

## 1. Introduction

Spontaneous isolated celiac artery dissection (ICAD) was infrequent and was described earliest by Foord AG and Lewis RD in 1959.^[1]^ The original reports on the topic always came from autopsy series which resulted in difficulty in understanding the clinical characteristics and made natural history studies impossible. With the application of high resolution computed tomography (CT) in clinic in recent years, more and more ICAD has been diagnosed and monitoring patients with dissections has become easier. The short-term complications of ICAD include organ ischemia necrosis and dissection rupture, while the long-term complications include dissection aneurysm rupture and hemorrhage, which can endanger life of patients.^[1–3]^ At present, the pathogenesis of ICAD is still unclear, and the best treatment is still controversial. Many reports suggested conservative treatment as optimal choice because the clinical symptoms of most patients can be relieved.^[4–6]^ However, the dissection progression and dissection aneurysm formation occurred after conservative treatment of some ICAD may make these patients miss the best intervention opportunity and increase the difficulty of later treatment.^[7,8]^

Spontaneous ICAD always have other visceral artery dissection, such as the dissection in superior mesenteric artery, left gastric artery (LGA), splenic artery, common hepatic artery (CHA), and gastroduodenal artery.^[9–13]^ Spontaneous ICAD and isolated superior mesenteric artery dissection (ISMAD) represent the dominant types of isolated visceral artery dissection (IVAD).^[14]^ Although it took up a proportion of 0.8% of all type of artery dissection,^[1]^ widespread application of computed tomography angiography improved the ability to diagnose ICAD and ISMAD at initial admission.

Though increasing studies reported the management of spontaneous ICAD, the characteristics and etiology of ICAD in different age-stage patients have not been well-studied. Thus, we performed a systematic review of the literature in order to compare information regarding the epidemiology, clinical profile and treatment of the spontaneous ICADs between the young and old patients.

## 2. Methods

### 2.1. Search strategy and study selection

We searched PubMed, Embase, and Web of Science up to March 1, 2023 for spontaneous ICAD case reports, using a search strategy developed by a medical information specialist that involved controlled vocabulary terms and related keywords: “celiac artery,”“visceral artery,” “dissecting,” “dissection.” The search was limited to English language articles. Two assessors independently screened the titles and abstracts of the studies. Full texts of relevant studies were obtained for further evaluation. Full texts of related references were also obtained for review. Reference lists of retrieved articles and relevant reviews were manually searched for additional studies. The ethical approval for this study was not applicable, for that it was a review and analysis of previous published reports.

### 2.2. Selection criteria

We included studies if they meet the following criteria: (1) patients diagnosed with ICAD; (2) case reports, case series, letters or editorials that reported the natural course, treatment, classification, and outcomes of ICAD; (3) studies reported detailed information about characteristics, treatment or outcomes of the cases.

Studies will be excluded if (1) patients suffered aortic dissection; (2) experimental trials on animals or nonhuman studies; (3) abstracts, expert opinions, reviews were excluded; (4) studies without sufficient data or did not meet our including criteria.

### 2.3. Data extraction

The information of each case was extracted from the included studies using a standardized extraction form. Key information of each case included sex, age, comorbidities/ history, symptoms, co-existing dissection, Sakamoto classification, extending of ICAD, complications, dissection length, distance from ostium, treatment, and outcome. Two authors independently collected data from included reports. Any disagreement was handled by discussion with a third reviewer.

### 2.4. Definition

The classification of spontaneous ICAD was defined as Sakamoto classification^[15]^ which based on the imaging appearance of the false lumen:

1. Type I: patent false lumen with both entry and reentry;
2. Type II: “‘cul-de-sac’”-shaped false lumen without reentry;
3. Type III: thrombosed false lumen with ulcer-like projection, which was defined as a localized blood-filled pouch protruding from the true lumen into the thrombosed false lumen;
4. Type IV: completely thrombosed false lumen without ulcer-like projection.

### 2.5. Data synthesis and statistical methods

We defined young patients as age < 50 years and old patients as age ≥ 50 years. Statistical analysis was performed using SPSS software (version 19.0; IBM Corp, Armonk, NY) and Stata 12.0 (Stata Corp., College Station, TX). Results are presented as the mean ± standard deviation or percent. Statistical significance was defined by 95% confidence intervals and *P* < .05.

## 3. Results

### 3.1. Included reports and cases

**Figure 1.**
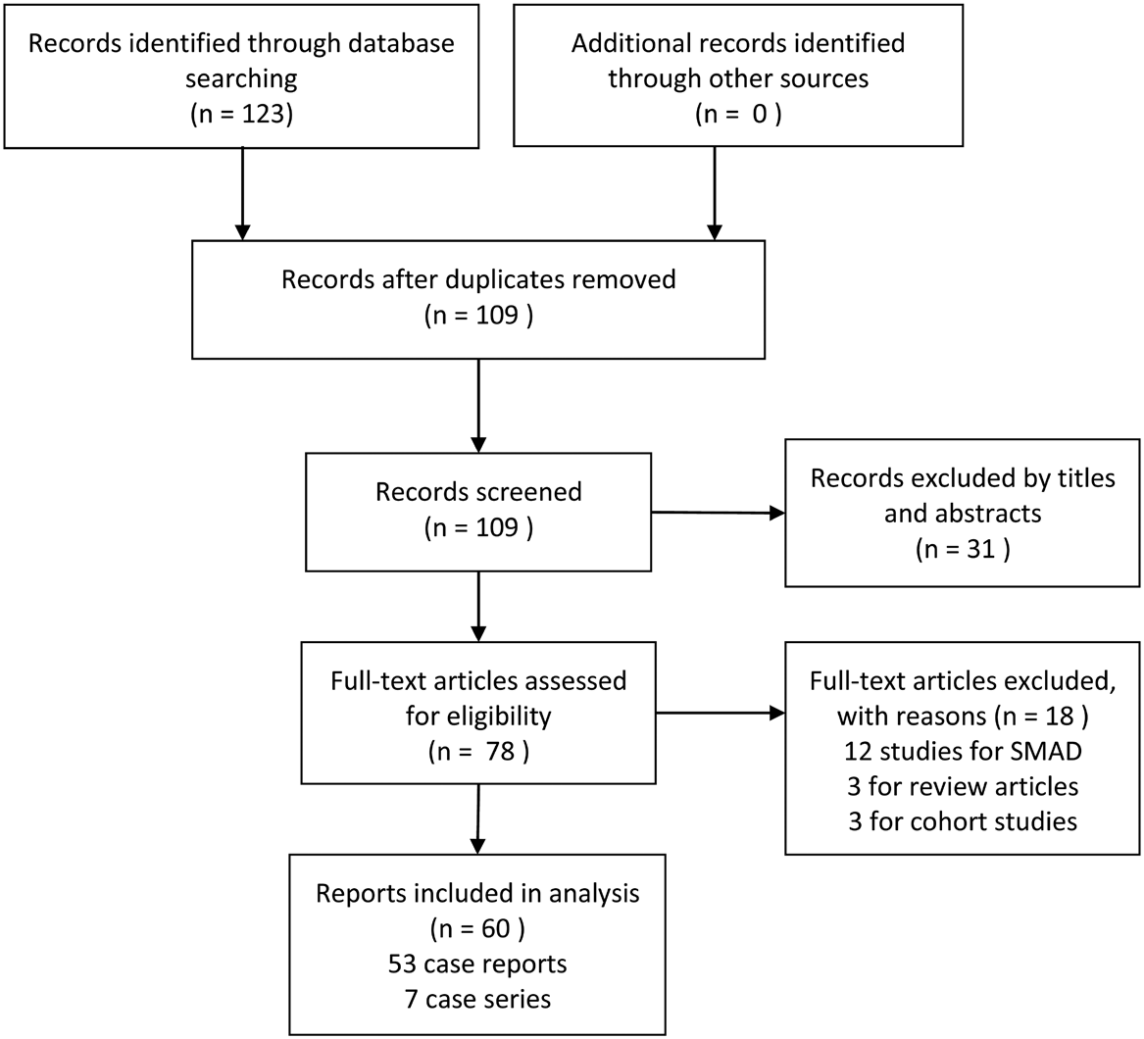
Flow diagram of literature search and selection of included studies for spontaneous ICAD case reports. ICAD = isolated celiac artery dissection.

A total of 123 records of citations were initially identified through database searching; after removing duplicates, 109 of records were reserved for further reviewing. 31 records were excluded via screening the titles and abstracts. Then 78 articles were assessed for eligibility. After reading the full texts, 18 studies were excluded further as displayed in Figure 1. Eventually, 60 reports (53 case reports^[2,3,16–66]^ and 7 case series^[7,11,67–71]^) with 125 ICAD patients were included for detailed information in analysis. According to our definition of age group, 62 cases were young patients and 63 cases were considered as old patients.

### 3.2. General demographics and clinical features of ICAD

The mean age was 52.4 years, with the majority being male (84.4%). In the 125 cases, 79 (63.2%) ICAD patients had comorbidities or history, with the top 2 conditions being hypertension (63.3%) and smoking (40.5%) respectively, following by dyslipidemia (10.1%) diabetes (6.3%) hyperuricemia (3.8%). The majority of patients were symptomatic and commonest presentation was abdominal pain (76.7%). In addition, many patients experienced abdominal pain accompanying with back pain (2.9%), chest pain (1.9%) and shock (1.9%). 4 (3.9%) and 3 (2.9%) ICAD patients presented with back pain and postprandial abdominal pain as main symptoms (Table 1).

**Table 1:** Demographic and clinical features of spontaneous isolated celiac artery dissection.

Demographic and clinical features	Sample size (n)	Mean ± SD/ %	Radiographic characteristics and complications	Sample size (n)	Proportion (%)
Age (year)	125	52.4 ± 10.7	*Co-existing dissection*		
*Sex*			SMAD	14	19.2
Male	103	84.4	Absent	59	80.8
Female	19	15.6	Sakamoto classification	32	
*Comorbidities/history*			I	6	18.75
Present	79	63.2	II	6	18.75
Hypertension	50	63.3	III	14	43.75
Smoking	32	40.5	IV	6	18.75
Dyslipidemia	8	10.1	*Extending of CAD*		
Diabetes	5	6.3	Absent	83	66.4
Hyperuricemia	3	3.8	Present	42	33.6
Other	22	27.8	CHA/ HA	33	78.6
Absent	32	25.6	SA	27	64.3
Unknown	14	11.2	LGA	4	9.5
*Symptoms*	125		GDA	2	4.8
Asymptomatic	4	3.2	*Complications*	29	
Symptomatic	103	82.4	SI	14	48.3
Abdominal pain	79	76.7	MH	4	13.8
AP with back pain	3	2.9	RH	3	10.3
AP with chest pain	2	1.9	MLD	2	6.9
AP with shock	2	1.9	SA rupture	2	6.9
Back pain	4	3.9	Other	4	13.8
Postprandial AP	3	2.9			
Other	10	9.7			
Unknown	18	14.4			

AP = abdominal pain, CAD = celiac artery dissection, CHA = common hepatic artery, GDA = gastroduodenal artery, HA = hepatic artery, LGA = left gastric artery, MH = mural hematoma, MLD = mild liver dysfunction, RH = retroperitoneal hemorrhage, SA = splenic artery, SI = splenic infarction, SMAD = superior mesenteric artery dissection.

### 3.3. Radiographic characteristics and complications of ICAD

Spontaneous ICAD always have other visceral artery dissection. Our results indicated that the main co-existing dissection was ISMAD (19.2%). Regarding to Sakamoto classification of ICAD, we found that type III was the most common classification of ICAD, with equal proportion of other classification. Spontaneous ICAD extended to distal arteries (33.6%) and the most common arteries extended were CHA or hepatic artery (HA) (78.6%), followed by splenic artery (64.3%), LGA (9.5%), and gastroduodenal artery (4.8%), respectively. Splenic infarction (48.3%) was the main complication of ICAD. The other common complications included mural hematoma (13.8%), retroperitoneal hemorrhage (10.3); mild liver dysfunction (6.9%), splenic artery (6.9%), and other (13.8%) (Table 1).

### 3.4. Treatment and outcomes of ICAD

The majority of ICAD patients (50.4%) received conservative treatment. Among them, anticoagulation treatment account for 44.4%, followed by antiplatelet (25.4%), anticoagulation combined antiplatelet (25.4%), and antihypertensive (4.8%). In addition, the proportion of patients receiving observation, endovascular and open surgery was 7.2%, 15.2%, and 4.8% respectively. Most cases showed no progression of dissection or aneurysm (71.7%) after the treatment and 17.0% cases experienced complete occlusion of the dissecting aneurysm during their follow-up (Table 2).

**Table 2:** Treatment and outcomes of spontaneous isolated celiac artery dissection.

Treatment	Sample size (n)	Proportion (%)
*Conservative*	63	50.4
Anticoagulation	28	44.4
Antiplatelet	16	25.4
Anticoagulation and antiplatelet	16	25.4
Antihypertensive	3	4.8
Observation	9	7.2
Endovascular	19	15.2
Open surgery	6	4.8
Conservative–endovascular	2	1.6
Open surgery and Endovascular	1	0.8
Unknown	2	1.6
*Outcomes*		
Complete occlusion of the dissecting aneurysm	9	17.0
No progression	38	71.7
Died	1	1.9
Improvement	3	5.7
Progression	2	3.8

### 3.5. Comparison between young and old ICAD regarding to demographic and clinical features

**Figure 2.**
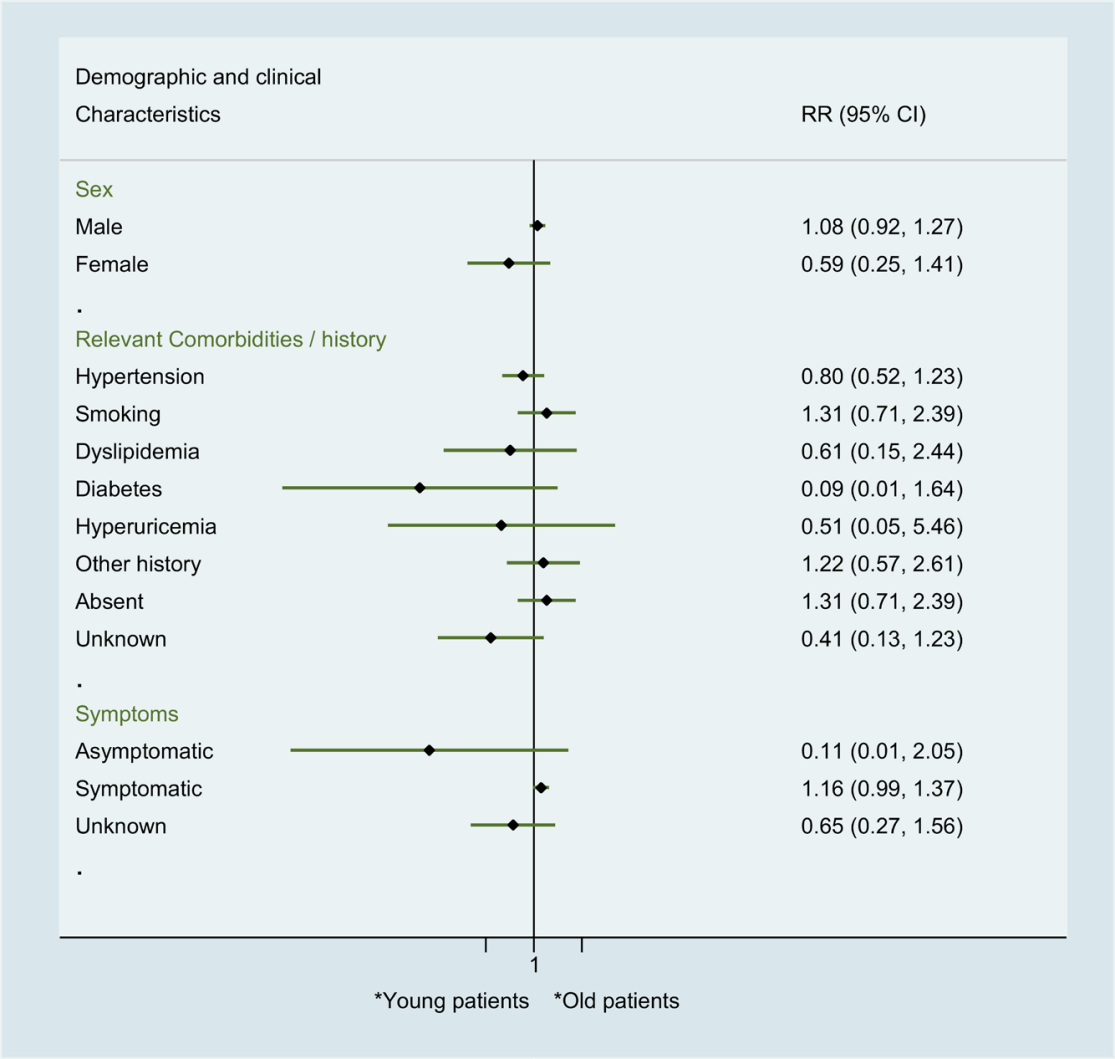
Comparison between young and old spontaneous ICAD patients regarding to demographic and clinical features. ICAD = isolated celiac artery dissection.

When comparing young to old patients with ICAD, no significant difference was found in demographic and clinical features including sex, comorbidities/ history (hypertension, smoking, dyslipidemia, diabetes, hyperuricemia), and symptoms (Fig. 2).

### 3.6. Comparison between young and old ICAD regarding to radiographic characteristics and treatment

**Figure 3.**
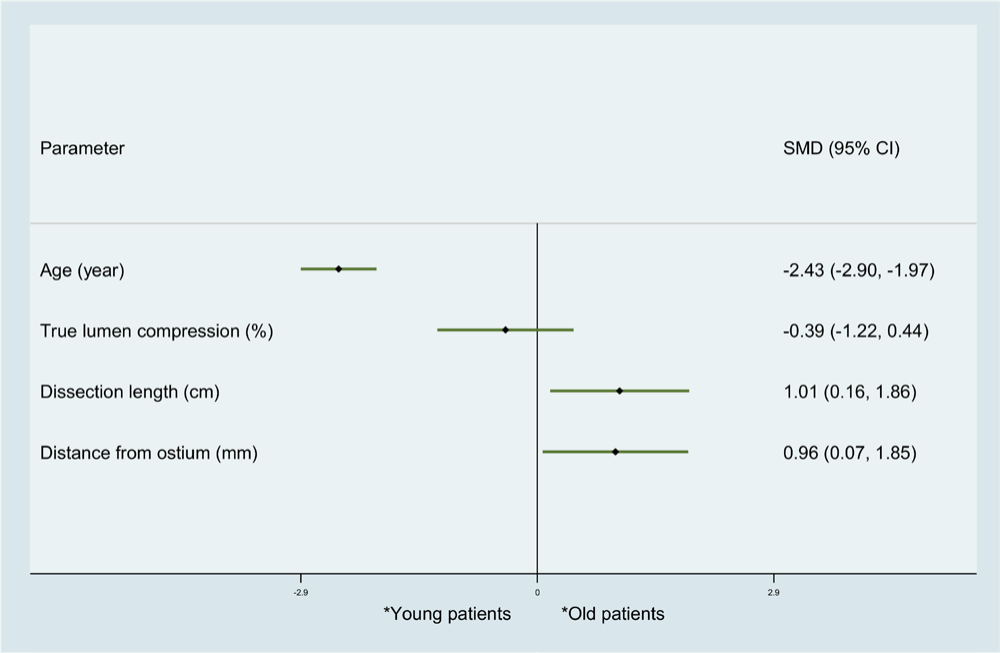
Comparison between young and old spontaneous ICAD patients regarding to age and dissection radiographic characteristics. ICAD = isolated celiac artery dissection.

**Figure 4.**
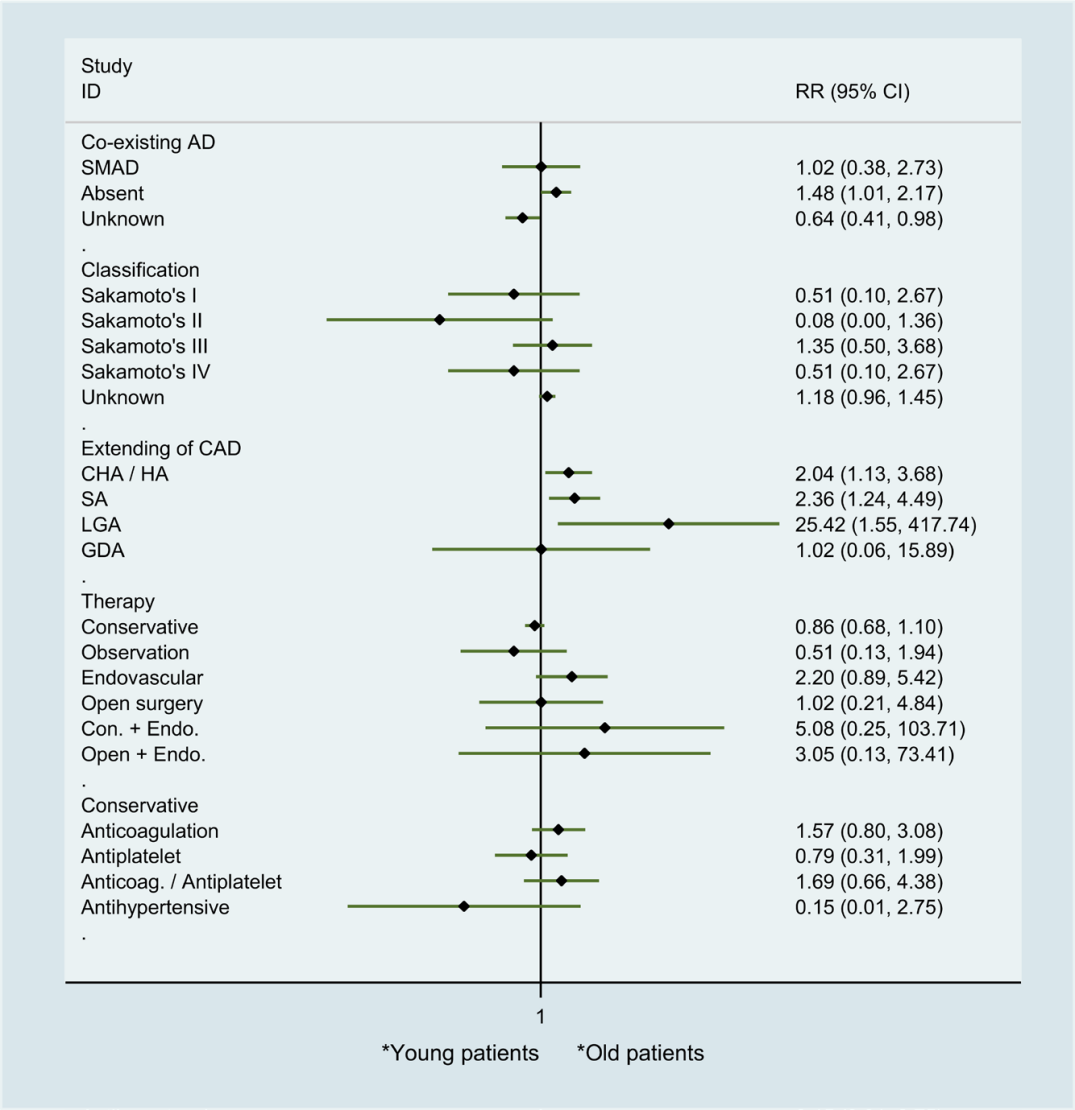
Comparison between young and old spontaneous ICAD patients regarding to radiographic characteristics and treatment. AD = artery dissection; CAD = celiac artery dissection; CHA = common hepatic artery; GDA = gastroduodenal artery; HA = hepatic artery; ICAD = isolated celiac artery dissection; ISMAD = isolated superior mesenteric artery dissection; LGA = left gastric artery; SA = splenic artery; endo. indicates endovascular; con. indicates conservative.

However, we found that young patients with ICAD experienced significantly longer dissection length (standardized mean difference [SMD] 1.01, 95% confidence intervals [CI] 0.16–1.86; *P* = .015) and distance from ostium (SMD 0.96, 95% CI 0.07–1.85; *P* = .013), but no significant difference was observed in true lumen compression (SMD −0.39, 95% CI −1.22–0.44; *P* = .364) (Fig. 3). In addition, our results also showed that ICAD in young patients extending more to distal arteries, including CHA/HA (risk ratio [RR] 2.04, 95% CI 1.13–3.68; *P* = .01), splenic artery (RR 2.36, 95% CI 1.24–4.49; *P* = .017) and LGA (RR 25.42, 95% CI 1.55–417.74; *P* = .04) (Fig. 4). However, we found no significant difference between young and old patients in co-existing artery dissection (RR 1.02, 95% CI 0.38–2.73; *P* = .09). Similarly, the distribution of Sakamoto classification in old and young ICAD patients was equal. Regarding to the treatment, no significant difference between young and old patients with ICAD was observed in conservation, observation, endovascular and open surgery (RR 1.02, 95% CI 0.21–4.84). For conservative treatment, our subgroup analysis showed similar proportion between young and old patients with ICAD in anticoagulation, antiplatelet, anticoagulation/antiplatelet and antihypertension (Fig. 4).

## 4. Discussion and conclusions

Spontaneous ICAD, defined as a dissection in the celiac artery without the involvement of the aorta, is a rare disease. Although symptomatic spontaneous ICAD patients are mostly diagnosed when they present with abdominal pain, some spontaneous ICAD patients are asymptomatic and can also be found incidentally on CT scanning or angiography.^[12,71]^ Most reports showed a middle aged male predominance in spontaneous ICAD patients.^[6,72,73]^

At present, the etiology behind spontaneous ICAD is unknown and is not well studied. Before our study, Cavalcante (2016) performed a systematic literature review of isolated spontaneous celiac trunk dissection (ISCTD) evaluating initial clinical and diagnostic aspects, treatment modalities and outcomes.^[73]^ The author identified a total of 169 patients and found that most common associated conditions were hypertension (31%) and smoking (23%).^[73]^ In addition, Wang J (2018) reviewed 8 studies investigating spontaneous ICAD.^[74]^ However, the study aimed to evaluating visceral artery dissection in both SMA and CA, which failed to clear the management profile of spontaneous ICAD.

Thus, our study included 125 ICAD patients whose detailed information was extracted from 60 previous reports. In addition, we grouped the cases by their age and compared the clinical features of spontaneous ICAD between young and old patients. According to the present study, the majority of ICAD patients received conservative treatment, but some need endovascular treatment or open surgery because of arterial rupture and hemorrhagic shock, rupture of the aneurysm.^[18,24,25]^ Given the rare previous reports included small numbers of patients, and data on its etiology, clinical features, management, hospital course, and outcomes were scarce. Spontaneous IVAD was reportedly observed in middle-aged men with comorbidities, such as hypertension and smoking,^[75,76]^ suggesting that IVAD might be associated with this factors. Similarly, spontaneous ICAD was also apt to middle-aged males. Hypertension and smoking were the top 2 conditions of ICAD patients. In addition, compared with other factors or history, diabetes and dyslipidemia which were in third and fourth place following hypertension and smoking, were commonly observed in ICAD patients. However, there was several significant difference between young and old patients regarding to radiographic characteristics: (1) young patients with ICAD experienced significantly longer dissection length and distance from ostium; (2) ICAD in young patients extending more to distal arteries, including CHA/HA, splenic artery and LGA. Thus, this result may explain our further results that young ICAD cases received more endovascular treatment but old IVAD patients received more observation, though there was no significant difference between young and old patients in treatments.

There were several deficiencies in our work. First, though we obtained large number of case data, it was very limited in many respects such as follow-up, long-term outcomes, operations and indicators for intervention etc. This limitation may lead to any risk of bias to our comparison results. Second, our case data were obtained from case reports or case series study which may lead to any report or publication bias. At present, no cohort study compared the difference between young and old patients with ICAD. Along with the development of medical examination technique and the application of high resolution CT in clinic, ICAD has been diagnosed and monitoring patients with dissections has become easier. Third, though we found significance of longer dissection length and distance from ostium in younger patients, this difference maybe resulted from our small sample size. Future researches could conduct more comprehensive data and could perform multivariate regression analysis which could clear the risk of factors of this dissection further.

In conclusion, though spontaneous ICAD had multitudinous clinic-pathologic features, it was apt to middle-aged males and symptomatic and abdominal pain was always the commonest presentation. Hypertension and smoking were the top 2 conditions of ICAD patients. However, there was significant difference between young and old ICAD cases regarding to radiographic characteristics which may indicated different pathogenesis and lead to different treatment or outcome between young and old ICAD case. Future prospective researches are needed and should focus on the different risk factors and long-term outcomes of ICAD in different age stage cases.

## Author contributions

**Conceptualization:** Tianbo Feng, Xiaoliang Chen.

**Data curation:** Xiaoliang Chen, Hongtao Wu, Shenghuan Wang.

**Formal analysis:** Tianbo Feng, Xiaoliang Chen.

**Methodology:** Hongtao Wu.

**Software:** Hongtao Wu.

**Writing – original draft:** Tianbo Feng, Xiaoliang Chen, Shenghuan Wang.

**Writing – review & editing:** Tianbo Feng, Xiaoliang Chen, Shenghuan Wang.
